# Recurrent ST-Elevation Myocardial Infarction (STEMI) in Coronary Artery Aneurysm Secondary to Atherosclerosis

**DOI:** 10.7759/cureus.28757

**Published:** 2022-09-03

**Authors:** Md Bhuiyan, Faraz Badar, Aqsa Ashraf, Emanuel D Chryssos, Asma Iftikhar

**Affiliations:** 1 Internal Medicine, Northwell Health, Mather Hospital, Pleasant Valley, USA; 2 Internal Medicine, Northwell Health, Port Jefferson, USA; 3 Internal Medicine, Northwell Health, Mather Hospital, Port Jefferson, USA; 4 Cardiovascular Medicine, Stony Brook University, Stony Brook, USA; 5 Pulmonary and Critical Care Medicine, Northwell Health, Mather Hospital, Port Jefferson, USA

**Keywords:** coronary artery angiography, artherosclerosis, dual-antiplatelet therapy (dapt), coronary aneurysm, st-elevation myocardial infarction (stemi)

## Abstract

We describe a rare case of coronary artery aneurysm (CAA) with recurrent ST-elevation myocardial infarction (STEMI) despite being on standard dual antiplatelet therapy (DAPT). A 47-year-old male presented with chest pain and was found to have inferior wall STEMI along with diffuse right coronary artery (RCA) ectasia and proximal RCA aneurysm, thrombotic occlusion, and dissection. He was managed with extensive thrombectomy, angioplasty, prolonged Heparinization, and DAPT. The patient went on to have a similar presentation nine months later with a recurrent inferior wall STEMI with proximal RCA aneurysm and thrombotic occlusion managed with thrombectomy and bare metal stent placement. He was placed on long-term anticoagulation and DAPT with no further recurrence of MI reported on follow-up.

## Introduction

A coronary artery aneurysm (CAA) is defined as a focal dilation of the coronary artery at least 1.5 times the adjacent normal segment. CAAs are rare, and most commonly caused by atherosclerosis [1]. They can be complicated by MI which is managed with Percutaneous Coronary Intervention (PCI), thrombectomy, stent placement, and DAPT. The role of anticoagulation is controversial in Acute Coronary Syndrome (ACS) with CAA. We describe a unique case of a 47-year-old male who suffered recurrent STEMI on DAPT, managed with PCI and subsequent long-term anticoagulation with no further reported MI episodes on follow-up.

## Case presentation

**Figure 1:**
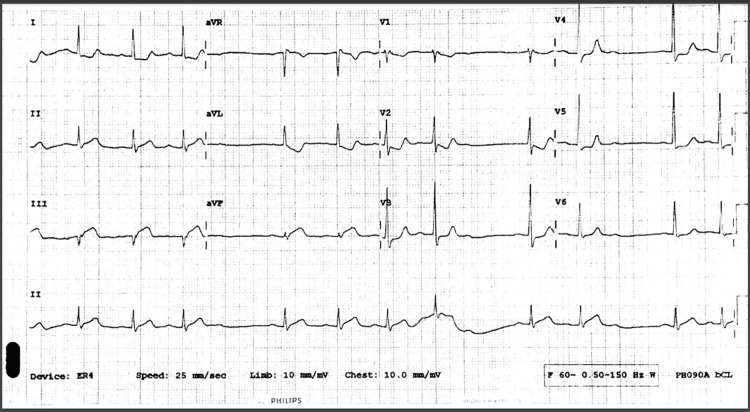
EKG demonstrating AV dissociation and ST elevations in leads II, III and aVF with reciprocal ST depression in aVL.

**Figure 2:**
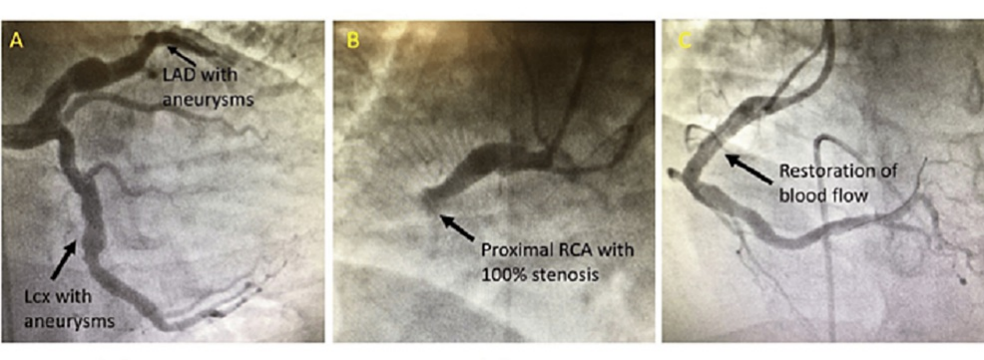
Cath demonstrating (A) LAD artery and circumflex artery with aneurysm, (B) proximal RCA with 100% stenosis and aneurysm, and (C) post-intervention restoration of blood flow in RCA. RCA: Right Coronary Artery, LAD: Left Anterior Descending

A 47-year-old non-smoker Greek male with a past medical history of hypertension and hyperlipidemia presented to the emergency department with sudden onset, progressively worsening non-radiating substernal chest pain of 8/10 intensity for one and half hours and was found to have inferior wall ST-elevation myocardial infarction (STEMI) on electrocardiogram (EKG) (Figure 1). Cardiac catheterization showed moderate ectasia and 40% stenosis in both the left anterior descending (LAD) and circumflex artery (Figure 2A). Cardiac catheterization also revealed diffusely ectatic right coronary artery (RCA) with dissection and heavy clot burden with 100% stenosis (Figure 2B). Mechanical thrombectomy was performed with balloon angioplasty (door to balloon time 37 minutes) and a significant clot burden was removed with an improvement in flow to RCA but no obvious flow to the right posterior descending artery (RPDA) and right posterior lateral branch (RPL). A significant size mismatch was noted from the distal RCA into these terminal branches. Serial balloon inflations were performed with some improvement in flow (Figure 2C). However, the distal vessels were inadequately opacified to clearly visualize their true anatomy. Due to ambiguity in vessel anatomy, significant clot burden, and size mismatch between the RCA and RPDA/RPL, PCI was aborted with no stent placement. The patient’s symptoms resolved within a day post-procedure, and he was discharged home on standard ACS medical therapy comprising aspirin 81mg daily, ticagrelor 90mg twice daily, atorvastatin 80mg daily, metoprolol 50mg daily, and valsartan 40mg at night.

On two-month follow-up, the stress test was normal, and the echocardiogram showed normal left ventricular function with an ejection fraction (EF) of 55%-60% which was slightly improved from 50% during hospitalization. Nine months later, the patient presented to the emergency department with similar symptoms and was found to have a second inferior wall STEMI. Cardiac catheterization revealed diffuse proximal RCA ectasia with 100% stenosis. Mechanical thrombectomy was performed with the addition of bare metal stent (BMS) placement this time. Post-intervention, there was a successful restoration of blood flow with the improvement of TIMI 0 to TIMI 3. Dual antiplatelet therapy (DAPT) had been adhered to and continued uninterrupted between the first and second MI. Anticoagulation was initiated with enoxaparin bridging to warfarin. Warfarin was continued long-term with a goal INR of 2-3.

The patient underwent an extensive workup to rule out other etiologies of CAA. C-reactive protein (CRP), antinuclear antibody (ANA) cascade, anticardiolipin, Jo-1, myeloperoxidase (MPO), and serine proteinase 3 (PR3) antibodies were all within normal limits. CT scans with the contrast of the neck, chest, abdomen, and pelvis were negative for the aneurysmal disease. An ultrasound of the aorta, iliac arteries, common femoral artery, superficial femoral artery, and the popliteal artery was also negative for an aneurysm. These normal studies ruled out vasculitides such as Kawasaki disease, Behcet’s disease, Takayasu arteritis, and other autoimmune and connective tissue disorders such as fibromuscular dysplasia.

**Figure 3:**
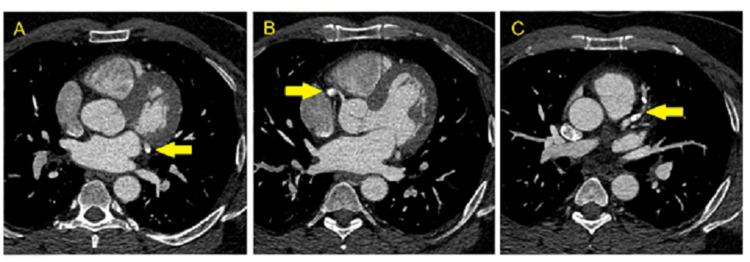
CTCA demonstrating (A) left circumflex artery with proximal plaque, (B) RCA with mid-segment focal restenosis and plaque, (C) LAD artery with ecstatic changes, mid, and distal plaques. CTCA: Computed Tomography Coronary Artery, RCA: Right Coronary Artery, LAD: Left Anterior Descending

CT coronary artery (CTCA) was performed six months after the second MI to monitor and analyze the extent of CAA. This showed LAD with ectatic changes, the proximal segment measuring 8mm, the mid-segment with 10%-25% calcified plaque, and the distal segment with 10%-25% non-calcified plaque. The RCA had mid-segment focal restenosis whereas the distal segment had 10%-25% non-calcified plaque. These CTCA findings with plaques indicated atherosclerosis as a possible etiology for our patient’s coronary aneurysm (Figures 3A-3C).

After the second MI, the patient remained on aspirin 81mg daily, ticagrelor 90mg twice daily, and warfarin for one year with no further report of significant cardiac events over six years upon follow-up.

## Discussion

A CAA is defined as a focal dilation of the coronary artery at least 1.5 times the adjacent normal segment. CAAs are rare and only observed in 0.3%-4.9% of patients undergoing coronary angiography [1-3]. They are predominant in males compared to females and more than 90% of adult CAAs are caused by atherosclerosis [1,4,5]. Smoking is another well-known risk factor for CAAs [6]. CAAs usually remain silent, however, can be complicated by thrombus formation or embolization leading to MI [5]. CAAs complicated by MI are managed with percutaneous coronary intervention (PCI), thrombectomy, stent placement, and DAPT. However, the role of anticoagulation is controversial in Acute Coronary Syndrome (ACS) with CAA. Recurrent MI from thrombosis in CAAs is rare. Our case report describes recurrent MIs in two different aneurysmal thrombotic sites. It should be noted that the second MI occurred while on DAPT. CAAs are most frequently noted in the proximal and middle segments of the RCA, about 40%-68% [1,5], and only 4.5% of CAAs involve both RCA and LAD [2]. Our patient had involvement in both proximal and distal RCA as well as LAD.

Patients with CAA having STEMI should undergo coronary angiography. Coronary angiography remains the gold standard for CAA diagnosis as it helps identify the shape, size, location, and co-existing anomalies such as coronary artery disease (CAD) [7]. Intravascular ultrasound can be helpful in providing details of vessel wall structure, size of CAA, and differentiating true aneurysm from pseudoaneurysm, which aids in proper stent sizing if PCI is planned. Noninvasive diagnostic modalities include coronary computed tomography and magnetic resonance angiograms.

In addition, further workup may be necessary to identify the possible etiology of CAA. These include lab tests such as CRP, ANA, anticardiolipin antibodies, Jo-1, MPO, and PR3 and imaging such as CT angiogram and arterial ultrasound to rule out vasculitis, autoimmune causes, and connective tissue disorders. Management of CAA varies depending on size, location, etiologies, and recurrent symptoms related to aneurysms [8]. Given the majority of the CAAs are associated with atherosclerosis, aggressive CAD risk factor modification is recommended. DAPT or therapeutic anticoagulation for CAA remains a subject of debate. As per our literature review, a consensus on the ideal choice of agent and duration of therapy is lacking. Coronary aneurysm complicated by MI frequently requires thrombectomy and DAPT due to the heavy clot burden. It was of utmost concern that our case developed recurrent thrombotic disease despite DAPT. Therefore, a decision was made to escalate therapy to include full anticoagulation with warfarin therapy. For the long-term, three antithrombotic agents were not preferred due to the risk of spontaneous bleeding, and the patient was maintained on dual antiplatelet and warfarin therapy to reduce his chances of recurrent thrombosis for 12 months. This decision was made based on individual provider experience and a detailed discussion with the patient weighing risks versus benefits.

For patients with recurrent MI, a combination of antiplatelet and anticoagulation had a better outcome in a few case studies but there is a lack of large-scale studies to support this data [5,9,10]. Doi et al. reported that despite having a higher risk of coronary artery ectasia (CAE) related cardiac events, patients with CAE treated with anticoagulation that achieved an optimal percent time in the target therapeutic range, defined as ≥60%, had no occurrence of major adverse cardiac events (P=0.03) versus patients without anticoagulation therapy or with percent time below target therapeutic range, defined as <60% [11].

In hindsight, failure to place a stent during the first PCI was likely the biggest culprit for recurrent thrombus development in our patient. It was a challenging procedure with the inability to achieve adequate flow despite multiple balloon inflations, inadequate visualization and opacification of distal vessels, significant clot burden, and size mismatch involving the RCA. All these were likely reasons that the first PCI was aborted with no stent placement. Although drug-eluting stents (DES) are now the norm for PCI, BMS still carries the potential advantage of safety in high risk of bleeding, especially if there is concomitant use of anticoagulation, as in our case [12]. A small number of cases report favorable outcomes with BMS placed for CAAs [13,14].

## Conclusions

In conclusion, CAAs are most commonly caused by atherosclerotic disease, and recurrent STEMI in them is rare. Even though there are no established treatment and follow-up guidelines currently, our case demonstrates that anticoagulation, in addition to standard management with PCI and DAPT, may be beneficial in such cases. Identification of etiology, morphology, and symptoms can help guide long-term treatment and follow-up.
